# Spatial bias in quality of estimated delivery date records in the electronic health record

**DOI:** 10.1093/jamiaopen/ooag183

**Published:** 2026-09-25

**Authors:** Abigail Lewis, Randi Foraker, Min Lian, Min Zhao, Inez Oh, Aditi Gupta, Antonina Frolova, Philip R O Payne

**Affiliations:** Institute for Informatics, Data Science, and Biostatistics, Washington University School of Medicine, St. Louis, MO 63110, United States; Department of Biomedical Informatics, Biostatistics, and Medical Epidemiology, University of Missouri School of Medicine, Columbia, MO 65201, United States; Division of General Medical Sciences, Department of Medicine, Washington University School of Medicine, St. Louis, MO 63110, United States; Institute for Informatics, Data Science, and Biostatistics, Washington University School of Medicine, St. Louis, MO 63110, United States; Institute for Informatics, Data Science, and Biostatistics, Washington University School of Medicine, St. Louis, MO 63110, United States; Institute for Informatics, Data Science, and Biostatistics, Washington University School of Medicine, St. Louis, MO 63110, United States; Division of Maternal—Fetal Medicine, Washington University School of Medicine, St. Louis, MO 63110, United States; Institute for Informatics, Data Science, and Biostatistics, Washington University School of Medicine, St. Louis, MO 63110, United States

**Keywords:** electronic health records, data accuracy, bias, spatial analysis

## Abstract

**Objectives:**

This study evaluates the quality of estimated delivery dates (EDD) captured in electronic health record (EHR) systems, focusing on identifying and quantifying bias in these estimates for patients seeking prenatal care.

**Materials and Methods:**

We measured spatial and racial biases in the completeness and concordance of EDDs recorded in the EHR for patients seeking prenatal care. Element presence and agreement were compared across the catchment area to identify spatial patterns of bias in EDD records.

**Results:**

We obtained 38 477 unique pregnancy episodes in the EHR representing 33 081 unique patients between 2018 and 2022. Clear spatial and racial biases emerged in the completeness and concordance of EDDs (*P* < .05 across tests): Black patients had higher levels of missing EDDs than White patients (8.43% vs 5.11%). Moreover, incompleteness and discordance in EDDs tended to occur in the same locations (*P* < .05 for all global tests), indicating a potential compounding effect on prenatal care access for the patients with implications for data reuse.

**Discussion:**

This work demonstrates spatial and racial biases in quality of EDDs recorded in the EHR and, therefore, the need to prioritize bias assessment within EHR data quality (DQ) frameworks. Continued efforts would support equitable improvements in EHR-based research by improving the reliability of current health service analyses and informing the development of more inclusive and effective EHR-based technologies.

**Conclusion:**

Including bias related to socioeconomic characteristics as a core axis of EHR DQ would extend existing frameworks to address potential inequities embedded in EHR data.

## Introduction

Electronic health record (EHR) systems facilitate healthcare delivery by systematically documenting interactions between patients and healthcare systems.^1^^,^^2^ The readily available nature of EHR data makes it a cost-effective and efficient resource for research to understand trends in healthcare utilization, patient outcomes, and effectiveness of care delivery.^3–5^ By leveraging these data, researchers can generate insights to improve patient care, optimize healthcare delivery, and advance population health.^5^

Despite their utility, EHR data are subject to limitations as they were designed for healthcare provision rather than research purposes. This misalignment often results in data quality (DQ) challenges that can preclude the reuse of EHR data for secondary applications.^6^^,^^7^ Extensive work has been conducted to assess and address EHR DQ, leading to development of multiple frameworks for evaluating the fitness of use in research.^8^^,^^9^ These frameworks typically encompass key dimensions of DQ, including completeness, correctness, concordance, currency, plausibility, conformance, and bias.^10^

Much of the existing literature on EHR DQ emphasizes functional assessments that determine whether the data are fit for use, sufficient for research, or accurate.^9^^,^^11^ While valuable, these assessments often overlook the causal factors underlying DQ issues and rarely incorporate ethical considerations. Recently, bias has emerged as a critical dimension of EHR DQ, offering insight into the social mechanisms that shape DQ.^10^ Disparities in DQ across socioeconomic groups may reflect biases embedded in broader systems of inequity. Evaluating such geospatial and sociodemographic biases in EHR DQ could help identify populations where DQ is most compromised, revealing communities that may be underserved or systematically overlooked by the healthcare system motivating interventions to improve equity in DQ. Given that sociodemographic and place-based factors influence healthcare utilization and outcomes, they are likely associated with individual-level variation in EHR DQ across many dimensions.^12^^,^^13^ Thus, bias may be better conceptualized by its interactions with other dimensions of DQ rather than defined as an independent dimension.

Prenatal care provides one setting in which high quality EHR data are necessary to ensure positive patient outcomes. Certain milestones of prenatal care should be met throughout the pregnancy in order to optimize obstetric outcomes and inadequate prenatal care can lead to adverse maternal and neonatal outcomes.^14–17^ EHR systems have been identified as a viable mechanism for improving prenatal care documentation and patient care provision, but have had uncertain reliability in obstetrics research purposes.^18–21^ A better understanding of EHR DQ in the context of prenatal care is needed to assess its reusability.

Prenatal care documentation in the EHR begins during an initial prenatal care appointment and continues throughout pregnancy. Initial pregnancy visits include recording the estimated delivery date (EDD) as a basis for tracking clinical milestones throughout the gestational period.^22^ Although EDDs can be updated, for example during early pregnancy based on ultrasound findings, incorrect or missing ADDs may hinder appropriate care provision. The quality of EDD recording in the EHR may impact both prenatal outcomes and EHR-based prenatal care research.

The objective of this study was to evaluate the quality of EDD recordings in the EHR for patients seeking prenatal care in a large health system in the Saint Louis, Missouri region, and to investigate spatial and racial biases in EDD recording quality. We specifically evaluated for spatial biases in completeness and concordance, which both have implications for patient care and data reuse. The results support practical recommendations for researchers and health systems to measure and address biases in EHR DQ.

## Methods

### Data

EHR data were extracted from the Barnes Jewish Christian (BJC) hospital system and Washington University School of Medicine for pregnancy episodes from June 1, 2018 to August 21, 2022. These data reflect all interactions between a patient and the BJC system during their pregnancy and included demographics, diagnoses, procedures, laboratory testing, and medications. Patient addresses were geocoded to a latitude/longitude coordinate using a protected health information secure instance of ArcGIS^23^ then matched to US Census regional boundaries at the US Census tract (CT) and county levels.^24^ This work was approved by the Washington University School of Medicine IRB (202001193) and participant consent was waived.

### Estimated delivery dates

EDDs are used in prenatal care to represent the expected end of the gestational period based on a pregnant patient’s presentation at an initial prenatal care visit. EDDs are determined through clinical assessment including menstrual cycle dating, ultrasound estimation, and others reflecting the biological definition of the prenatal period (Table 1). After assignment, EDDs guide prenatal care delivery by supporting proper timing of care and accuracy of diagnoses throughout the prenatal period.^25^^,^^26^ For this study, EDDs were extracted from structured EHR (Epic) data. Changes in EDDs may reflect meaningful clinical developments or record errors requiring modification. For pregnancy episodes with multiple EDD entries, we defined the EDD range as the number of days between the earliest and latest EDDs, and the dating range as the number of days between the earliest and latest dates on which EDDs were entered. The EDD range represented the magnitude of EDD disagreement within an episode while the dating range represented time-to-stabilization.

**Table 1.** Observation count of dating event types.

Dating event type	Observation count
Alternate EDD entry	9346 (19.85%)
Bedside ultrasound	45 (0.10%)
Day 3 embryo transfer	14 (0.03%)
Day 5 embryo transfer	245 (0.52%)
Embryo Transfer	69 (0.15%)
Est. date of conception	319 (0.68%)
Interfaced EDD	69 (0.15%)
Last menstrual period	15 142 (32.16%)
Other basis	3316 (7.04%)
Patient reported	5909 (12.55%)
Ultrasound	10 079 (21.41%)
Uterus size	8 (0.02%)
Missing (NULL)	2518 (5.35%)

*N* = 47 079 across 38 477 episodes.

### Data quality analysis

**Figure 1.**
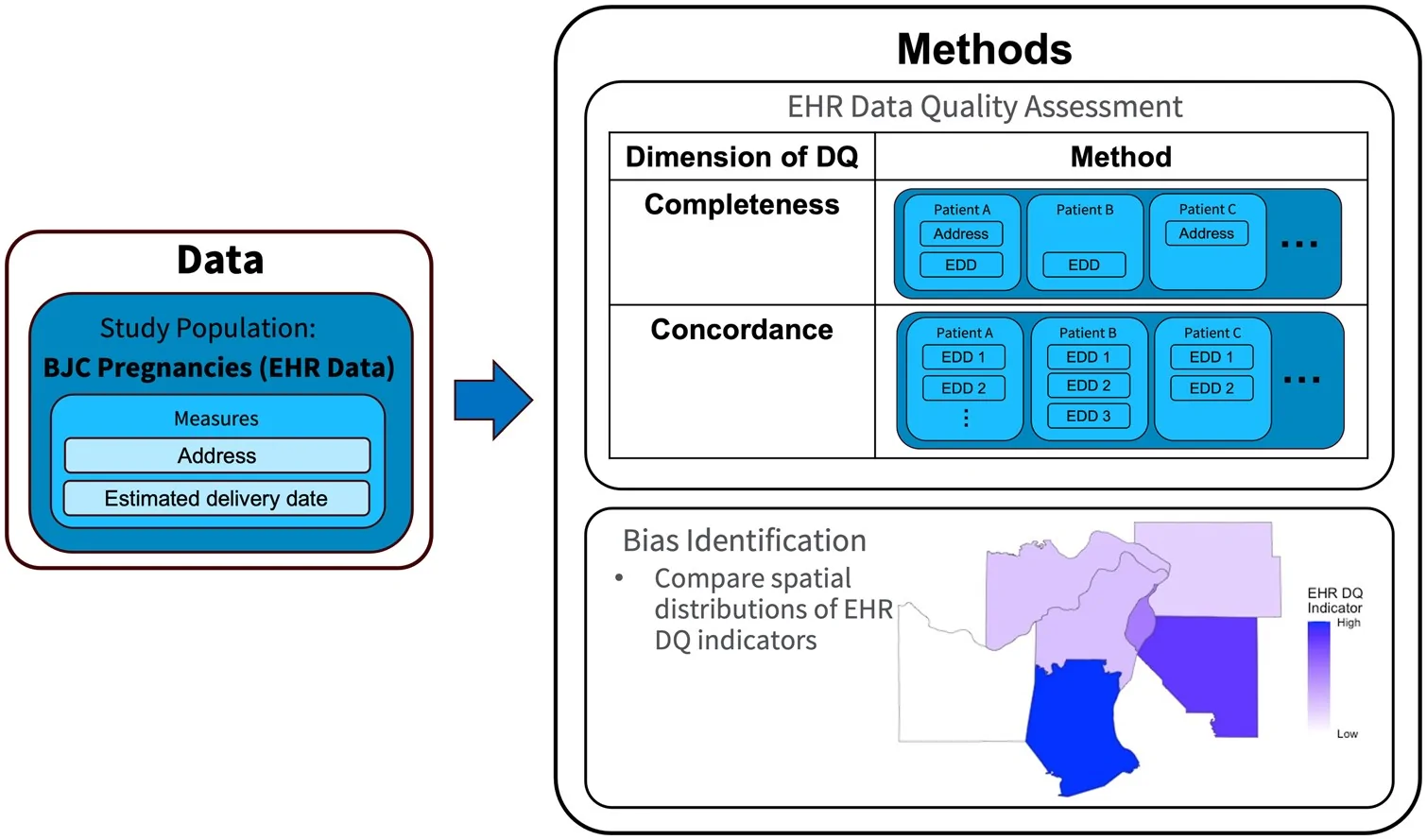
Workflow diagram.

All analyses were completed using pregnancy episodes as the unit of analysis; unique patients could have multiple episodes in the study time window. Inclusion and exclusion criteria were defined individually by DQ measure to reflect relevance to the measure. Traditional inclusion and exclusion criteria may differentially remove patients in a manner reflecting DQ issues, which could skew or minimize the exact DQ outcomes we intended to measure. All analyses were completed in R.^27^ We assessed for bias in the completeness and concordance of EDDs recorded in the EHR. The full workflow can be found in Figure 1.

#### Measures of completeness

In EHR DQ analyses, completeness refers to the presence of elements in the EHR and is most often assessed by element presence.^8^^,^^10^ We assessed the completeness of patient addresses and EDD by measuring element presence across the cohort of interest. Element presence determines whether elements of interest are available in the EHR. Analyses were first completed for the entire cohort, and then were stratified by race, location, and their intersection to assess for bias in completeness levels. Tests were repeated restricting to one pregnancy per patient to address independence assumptions.

To investigate racial bias in completeness, we used chi-square tests to compare element presence count distributions across racial groups. To investigate spatial bias in EDD completeness, we first compared element presence by county using a chi-square test. We then used generalized additive models (GAM) with thin plate regression splines to measure the local odds of a patient having an EDD and plotted the odds ratio and significance overlays based on the covariance matrix of the smoothing coefficients for each location, with the overall odds of having an EDD as the baseline measure.^28^

#### Measures of concordance

In EHR DQ analyses, concordance refers to agreement between elements within the EHR or between elements in the EHR and another data source.^8^^,^^10^ We specifically explored concordance of EDDs within the EHR using data element agreement. Indicators included the number of recorded EDDs, the EDD range for an individual episode, and the dating range for individual episodes. Discordant EDDs were identified by EDD and dating ranges. These measures were evaluated for the entire cohort and for cohorts stratified by race. Racial biases were identified using the Wilcoxon rank-sum (WRS) test. To identify spatial biases in the above measures, we used GAMs with a quasipoisson (log) link function and thin plate regression splines. A quasipoisson model was used to address overdispersion in ranges. As a sensitivity analysis, we compared EDD and dating ranges across dating source trajectories to distinguish between EDD changes that were likely clinically validated vs true DQ deficiencies.

#### Spatial analyses

**Figure 2.**
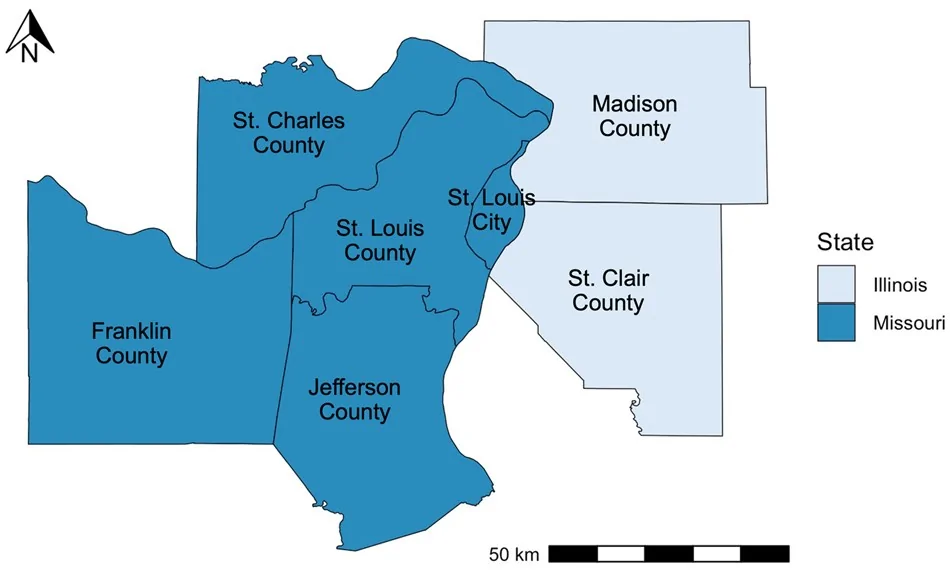
Map of counties in St. Louis area.

Spatial analyses were limited to the 7 counties representing a circle with a 20 mile radius around the St. Louis area which included Madison County [FIPS: 17119], St. Clair County [FIPS: 17163], Franklin County [FIPS: 29071], Jefferson County [FIPS: 29099], St. Charles County [FIPS: 29183], St. Louis County [FIPS: 29189], and St. Louis City [FIPS: 29510]. This region encompassed most of the St. Louis region and most of the pregnancy episodes in the study (Figure 2).

## Results

We extracted 38 477 pregnancy episodes representing 33 081 unique patients in the study period. Of those, 25 893 episodes were included in spatial analysis based on the criteria in the Methods.

### Completeness

Of the 38 477 unique episodes, 6248 (16.24%) were either missing an address or had an address that could not be geocoded leading to addresses being 83.76% complete across all episodes. Addresses could not be geocoded when they were incorrectly recorded, included a unit number with no corresponding street address, referred to a nonexistent street, or when they referenced a PO or RR box. The percentage of complete addresses was significantly different between patient races both when considering all episodes and when restricted to one episode per patient (chi-square = 53.8 [df = 2], *P* < .001, and chi-square = 54.1 [df = 2], *P* < .001 respectively). However, the difference in address completeness between Black and White patients was only 1.6% which is likely not clinically significant. The highest percentage of incomplete addresses occurred for patients who did not identify as Black or White followed by patients who identified as Black (Table 2). 2518 (6.54%) episodes were missing an EDD resulting in 93.46% complete EDDs across all episodes. The percentage of complete EDDs varied significantly by race such that Black patients were most likely to be without an EDD (8.43% vs 5.51% for White patients and 7.72% for Other patients, chi-square = 156.2 [df = 2], *P* < .001). This result remained when only including one episode per patient (7.97% for Black patients, 4.58% for White patients, 7.33% for Other patients, chi-square = 155.5 [df = 2], *P* < .001).

**Table 2.** Completeness by race.

Variable	All episodes (*N* = 38 477)	Race			
		Black or African American (*N* = 11 758)	White (*N* = 23 172)	Other (*N* = 3306)	Chi-square Test
Address					
Address	32 229 (83.76%)	9786 (83.23%)	19 654 (84.82%)	2648 (80.10%)	chi-square = 53.82 [df = 2], *P* < .001
No address	6248 (16.24%)	1972 (16.77%)	3518 (15.18%)	658 (19.90%)	
EDD					
EDD	35 959 (93.46%)	10 766 (91.56%)	21 988 (94.89%)	3051 (92.29%)	chi-square = 156.2 [df = 2], *P* < .001
No EDD	2518 (6.54%)	992 (8.43%)	1184 (5.11%)	255 (7.72%)	

**Unique patients**					

**Variable**	**All episodes (*N* = 33 081)**	**Black or African American (*N* = 9979)**	**White (*N* = 19 849)**	**Other (*N* = 3016)**	**Chi-Square test**

Address	26 833 (81.11%)	8293 (83.10%)	16 823 (84.75%)	2404 (79.71%)	chi-square = 54.1 [df = 2], *P* < .001
No address	5424 (18.89%)	1686 (16.90%)	3026 (15.25%)	612 (20.29%)	
EDD					
EDD	31 068 (93.91%)	9184 (92.03%)	18 939 (95.42%)	2795 (92.67%)	chi-square = 150.5 [df = 2], *P* < .001
No EDD	2013 (6.09%)	795 (7.97%)	910 (4.58%)	221 (7.33%)	

**Figure 3.**
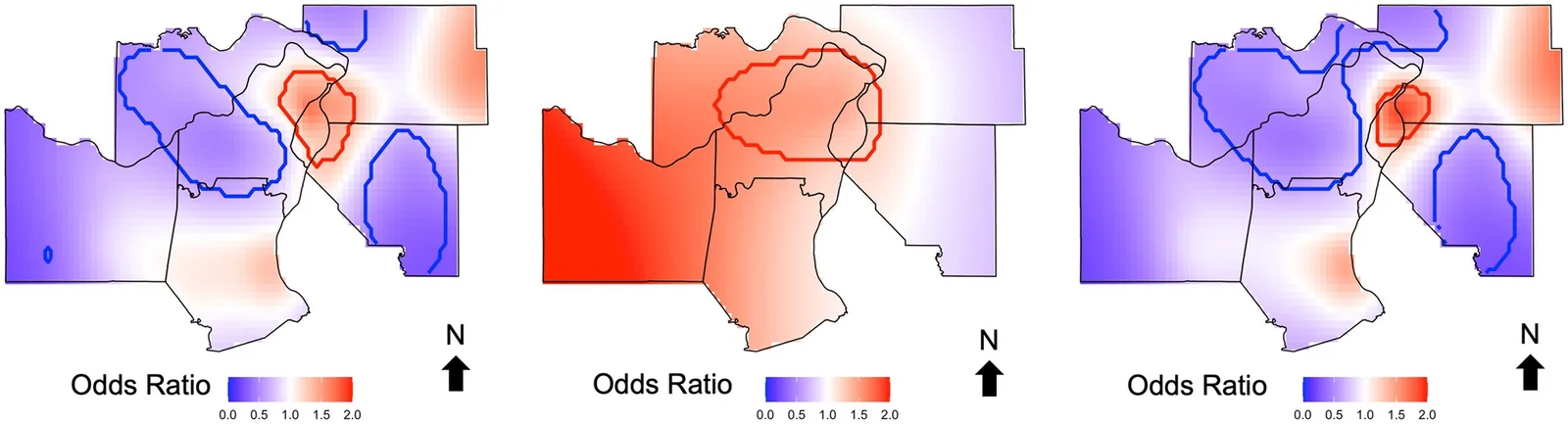
Spatial distribution of odds of EDD incompleteness in the EHR. Maps are centered around St. Louis City and St. Louis County. Red areas indicate high odds of EDD incompleteness while blue areas indicate low odds of EDD incompleteness for the 7 included counties. Red lines show significant regions of low odds and blue lines show significant regions of high odds. Left: all patients. Middle: Black patients. Right: White patients.

In the cohort eligible to be included in spatial analyses, 1655 (6.39%) episodes had an incomplete EDD. We first identified spatial variation in EDD completeness at the county level using a chi-square test (All epsiodes: chi-square = 67.2 [df = 6], *P* < .001, one episode per patient: chi-square = 59.1 [df = 6], *P* < .001). After positive identification of spatial patterns, we proceeded with more detailed GAM analyses. Figure 3 shows regions of high (red) or low (blue) odds of EDD incompleteness. EDD incompleteness tended to occur around Saint Louis City extending into northern parts of Saint Louis County, western parts of Illinois, and southern portions of Missouri. EDD incompleteness tended to occur in the northern half of Saint Louis City and County and most of the counties in Illinois. Patterns of EDD completeness differed when stratified by race (Figure 3). The spatial pattern of incompleteness of EDDs of White patients (Figure 3, right) as similar the full cohort (Figure 3, left), while the spatial pattern of EDD incompleteness of Black patients appears inverse that of White patients. However, we also identified a statistically significant intersection of EDD and address completeness (all episodes: chi-square = 22.90 [df = 1], *P* < .001, one episode per patient: chi-square = 32.3 [df = 1], *P* < .001) in which patients missing an address were more likely than patients with an address to be missing an EDD as well (all episodes: 19.66% vs 16.00%, one episode per patient: 20.93% vs 16.1%). Thus, by removing individuals without a geocodable address from the spatial analyses, we may be underestimating the observed spatial biases.

### Concordance

We extracted 47 079 EDD observations for 38 477 unique episodes. Each unique episode had up to 12 recorded EDDs, with most episodes having one (29,194; 75.25%) or two (5595; 14.44%) recorded EDDs (Table 3). EDDs were most often dated using the last menstrual period (15 142; 39.08%), ultrasound (10 079; 26.01%), or an alternate EDD entry (9346; 24.12%) (Table 1). A total of 6765 episodes had multiple EDD observations. For the remaining concordance analyses, we only included the 6765 episodes with multiple EDDs.

**Table 3.** Number of EDD observations per episode.

Number of EDD observations	Number of episodes *N* = 38 477	Episodes of Black patients *N* = 11 758	Episodes of White patients *N* = 23 172
0	2518 (6.54%)	992 (8.43%)	1184 (5.11%)
1	29 194 (75.35%)	8440 (71.78%)	18 211 (78.59%)
2	5595 (14.44%)	1956 (16.63%)	3140 (13.55%)
3	899 (2.34%)	345 (2.93%)	480 (2.07%)
4	193 (0.50%)	67 (0.56%)	114 (0.49%)
5	51 (0.13%)	23 (0.19%)	23 (0.10%)
6	14 (0.04%)	3 (0.03%)	11 (0.05%)
7	7 (0.02%)	2 (0.02%)	4 (0.02%)
8	4 (0.01%)	0	3 (0.01%)
9	1 (0%)	0	1 (0%)
12	1 (0%)	0	1 (0%)

**Figure 4.**
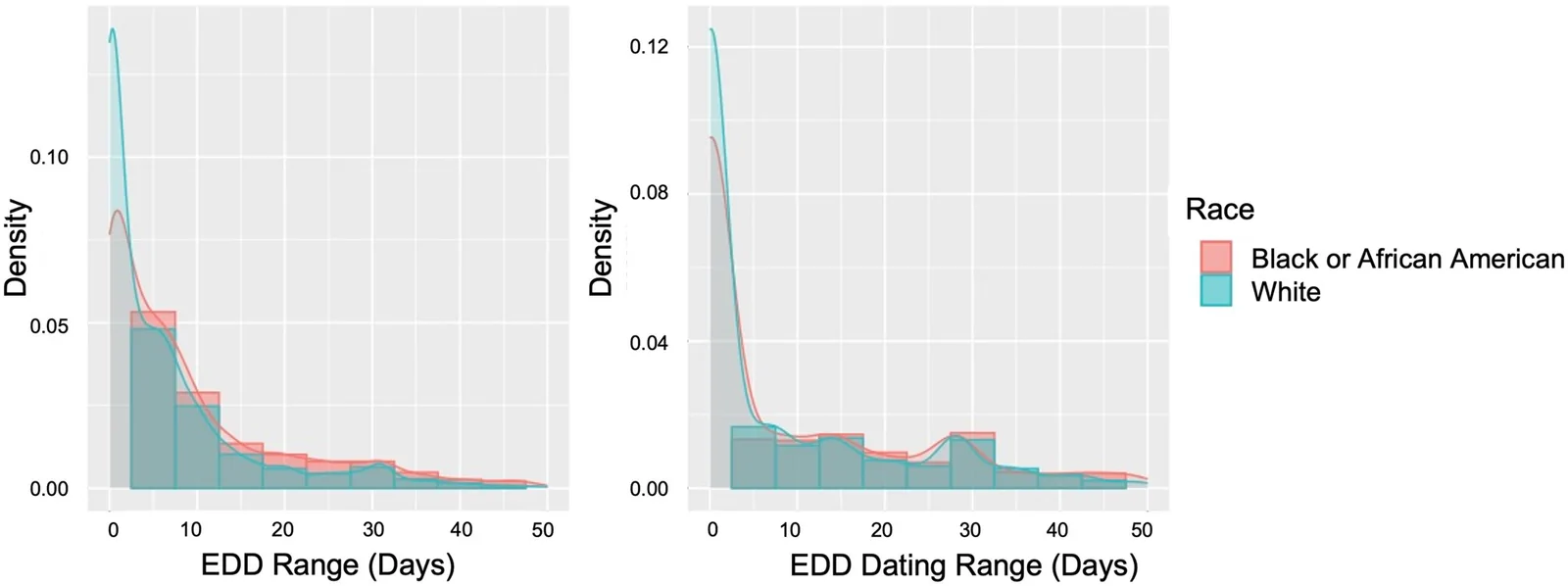
Histogram and density plots of concordance indicators. Left panel: EDD ranges. Right panel: EDD dating ranges.

EDD ranges were skewed right and spanned from 0 to 12 053 days (Q1: 1, Q2: 5, Q3: 13). 93 episodes had recorded EDDs at least a year apart. As shown in Figure 4, when stratified by race, White patients on average had smaller EDD ranges than Black patients. EDD ranges of Black patients spanned from 0 to 8382 days (Q1: 1, Q2: 6, Q3: 18), while EDD ranges of White patients spanned from 0 to 12 053 days (Q1: 1, Q2: 4, Q3: 11). Although WRS tests for episodes with multiple EDD entries was significant (*P* < .001), 95% confidence interval ranged from 1 to 2 days showing likely non-clinically meaningful differences in ranges. However, Black patients in the fourth quartile of EDD ranges experienced differences of a week more than White patients in the fourth quartile of EDD ranges (18 vs 11 days) suggesting that differences in the skewness of EDD ranges may lead to clinically meaningful differences care delivery for patients at the high end of the distribution.

Dating ranges spanned from 0 days to 386 days (Q1: 0, Q2: 4, Q3: 30). When stratified by race, White patients had a smaller EDD dating range on average than Black patients; the density of Black patients as slightly larger than the density of White patients as the time range increased (Figure 4). However, the differences were less extreme than the patterns in EDD ranges. Dating ranges for Black patients spanned from 0 to 342 days (Q1: 0, Q2: 7 days, Q3: 35 days), while dating ranges of White patients spanned from 0 to 386 days (Q1: 0, Q2: 3, Q3: 28). Again, the WRS test for episodes with multiple EDD entries returned a non-clinically meaningful confidence interval (1.6 × 10^−6^ to 4.2 × 10^−5^ days) despite a significant *P*-value (*P* < .001). But similarly to EDD ranges, Black patients in the fourth quartile of EDD dating ranges experienced difference of a week more than White patients in the fourth quartile of EDD ranges (35 vs 28 days).

**Figure 5.**
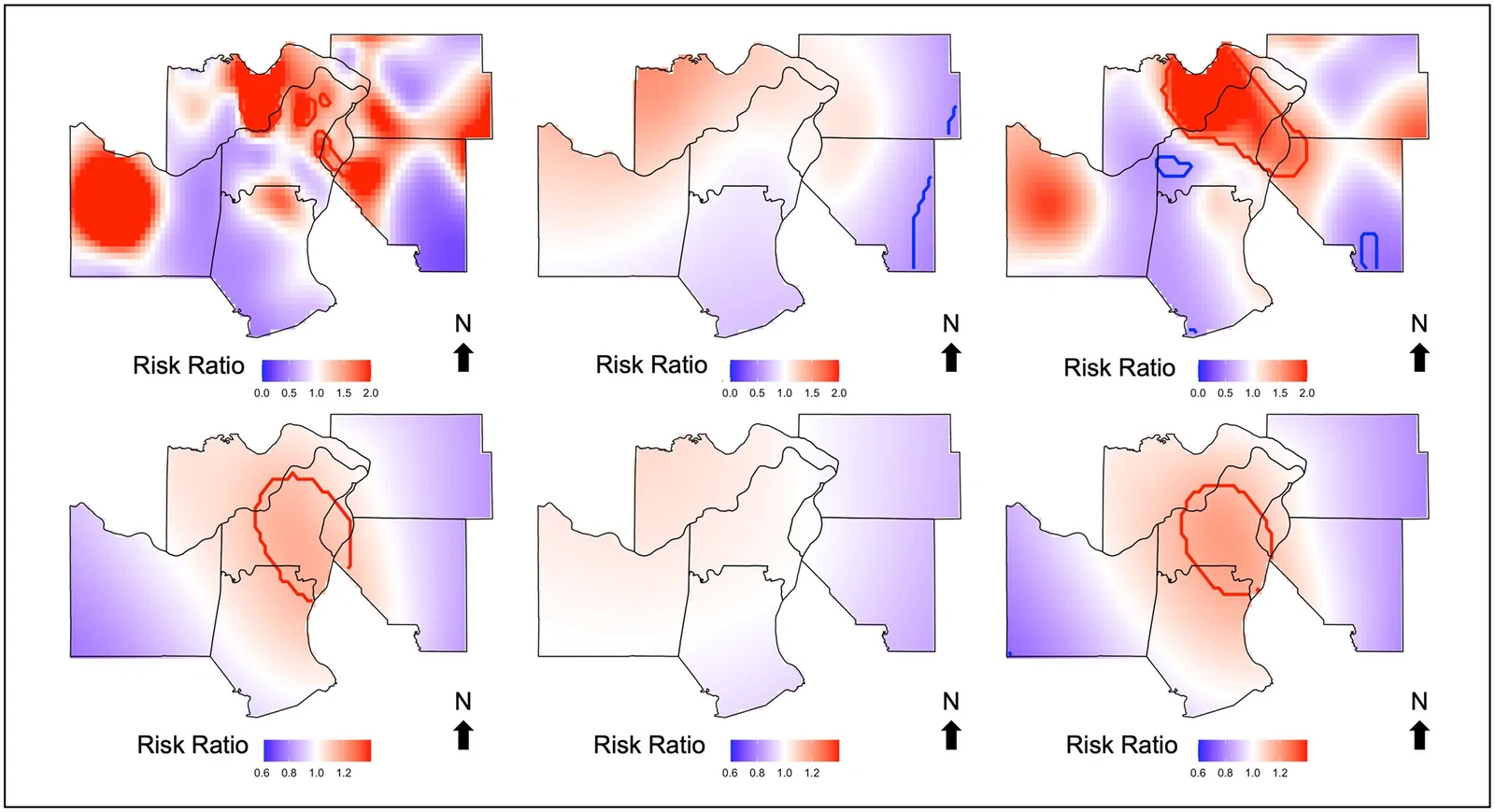
GAM for concordance ranges. Maps are centered around St. Louis City and St. Louis County. All panels use a span of 0.15. Red areas indicate longer ranges, while blue areas indicate shorter ranges for the 7 included counties. Red lines show significant regions of longer ranges and blue lines show significant regions of lower regions. Maps include episodes with multiple EDDs. Top row: EDD ranges. Bottom row: dating ranges. Left column: all patients. Middle column: Black patients. Right column: White patients.

The top row of Figure 5 shows the spatial patterns of risk of increased EDD ranges for episodes with multiple EDDs. We identified a clear spatial distribution in EDD ranges. Most of the City of Saint Louis and northern Saint Louis County had a higher risk of increased EDD ranges, while west Saint Louis County had a decreased risk of longer EDD ranges. The bottom row of Figure 5 shows the spatial patterns of risk of increased EDD dating ranges for episodes with multiple EDDs. We found spatial patterns in dating ranges suggesting there was spatial variation in the time it takes to determine a correct EDD.

Table 4 shows EDD ranges and dating ranges across various sequential combinations of EDD dating sources. While some trajectories like last menstrual period followed by ultrasound present clinically reasonable EDD ranges of 15 days, other trajectories like Alternate EDD Entry to Alternate EDD Entry show extended EDD ranges of 71 days without providing any indication of clinical reasoning.

**Table 4.** EDD range and dating range by EDD dating sources for episodes with multiple EDDs (*N* = 6765).

EDD dating source trajectory	Number of episodes	EDD range mean (SD)	EDD dating range mean (SD)
Last menstrual period -> Ultrasound	1278 (18.89 %)	15.30 (23.54)	24.75 (37.28)
Alternate EDD entry -> last menstrual period	515 (7.61 %)	5.39 (28.50)	20.66 (45.22)
Alternate EDD entry -> Alternate EDD Entry	508 (7.51 %)	70.93 (666.61)	19.30 (36.21)
Last menstrual period -> Last menstrual period	470 (6.95 %)	33.90 (87.74)	25.94 (55.59)
Alternate EDD entry -> Ultrasound	418 (6.18 %)	10.59 (27.76)	21.40 (42.10)
Ultrasound -> Last menstrual period	325 (4.80 %)	10.00 (23.04)	21.62 (47.79)
Ultrasound -> Ultrasound	319 (4.72 %)	20.11 (80.11)	21.97 (44.05)
Last menstrual period -> Alternate EDD entry	257 (3.80 %)	6.26 (23.95)	9.74 (28.59)
Patient reported -> Patient reported	226 (3.34 %)	108.42 (172.85)	15.14 (29.91)
Ultrasound -> Alternate EDD entry	163 (2.41 %)	11.36 (33.28)	13.15 (40.98)
Other basis -> Other basis	144 (2.13 %)	52.28 (116.37)	17.58 (37.84)
Patient reported -> Last menstrual period	121 (1.79 %)	9.76 (36.88)	20.86 (33.39)
Last menstrual period -> Last menstrual period -> Ultrasound	116 (1.71 %)	38.72 (76.67)	33.25 (44.19)
Last menstrual period -> Patient reported	114 (1.69 %)	6.38 (18.38)	81.63 (83.62)
Last menstrual period -> Other basis	86 (1.27 %)	12.21 (25.46)	54.13 (68.85)
Alternate EDD Entry -> Patient Reported	81 (1.20 %)	8.27 (25.41)	58.19 (70.41)
Alternate EDD entry -> Other basis	69 (1.02 %)	9.23 (17.34)	48.04 (57.76)
Patient reported -> Ultrasound	67 (0.99 %)	3.22 (6.48)	24.78 (39.54)
Alternate EDD entry -> Alternate EDD entry -> Alternate EDD entry	54 (0.80 %)	69.42 (117.96)	37.06 (53.32)
Last menstrual period -> Last menstrual period -> Last menstrual period	52 (0.77 %)	78.66 (138.37)	52.12 (75.20)
Last menstrual period -> Ultrasound -> Ultrasound	52 (0.77 %)	21.85 (54.01)	31.87 (46.54)
Other basis -> Ultrasound	51 (0.75 %)	5.39 (8.18)	23.16 (37.94)
Ultrasound -> Patient reported	50 (0.74 %)	4.67 (17.16)	77.39 (78.81)

EDD dating source trajectories show sequential dating sources. Trajectories occurring in fewer than 50 episodes were excluded from this table due to their limited occurrences.

## Discussion

The objective of this study was to evaluate the quality of EDD recordings in the EHR for patients receiving prenatal care through the BJC HealthCare system in St. Louis. We identified DQ issues in the dimensions of completeness and concordance that varied across patients’ race and home location, indicating differential DQ levels. These DQ issues have critical implications for secondary data use in prenatal care research. Pregnancy dating relies on EDDs, which in EHR-based studies often serve as the sole temporal reference for defining outcomes of interest such as adherence to prenatal care visits, preterm birth, or other pregnancy related measures. Although not always explicitly stated, EDDs are frequently required for inclusion in EHR based cohorts suggesting that incomplete EDD documentation could lead to removal from real-world research cohorts. Additionally, discordant EDDs could lead to misclassification of pregnancy timing, producing biased estimates of exposures, outcomes, or care utilization through ambiguous or incorrect pregnancy dating.

Further complicating prenatal care DQ were racial and spatial biases. Black patients had higher levels of incomplete and discordant data than White patients, indicating racial inequities in EHR DQ. Although confidence intervals from WRT tests showed little clinical utility, quartile comparisons showed larger discordance by a week for Black patients which could substantially impact cohort definitions, outcome classification, or care delivery through delayed or lack of intervention or rendering patients ineligible for screening opportunities. These DQ issues were not randomly distributed geographically but often occurred in the same geographic locations, potentially compounding their impact on downstream analyses. The lower proportion of patients with geocodable addresses and EDDs may have led to underestimation of the observed spatial biases. The observed patterns reflect a larger system of disparate healthcare access, and specific geographic distributions of inequity in the St. Louis region, suggesting that data reuse implications may disproportionately impact certain populations.^13^^,^^29^^,^^30^

Multiple factors may contribute to these disparities, including care-seeking behaviors of patients, barriers to adequate prenatal care, variation in clinical documentation practices, or meaningful clinical developments. In prenatal care, Black patients are more likely than White patients to initiate care in the second or third trimester, complicating EDD estimation.^31–35^ Barriers such as insurance access, provider availability, transportation, and cost also affect the timeliness and continuity of care and associated completeness and concordance.^36^^,^^37^ In addition, prenatal care is often fragmented across institutions, as high-risk pregnancies may be transferred to tertiary centers late in pregnancy or delivery and prenatal care may occur within different health systems.^38^^,^^39^ These care transfers may lead to clinically truthful EDD or dating ranges thus conflating poor DQ with poor care access still presenting avenues for improvements in prenatal care delivery.

Regardless of their source, biases in EHR DQ must be addressed to ensure patients receive appropriate care. In the context of EHR data reuse, biased EDD completeness could lead to the differential exclusion of populations from secondary analyses, while discordant EDDs may result in systematic misclassification, both of which have implications on validity and generalizability for excluded or incorrectly characterized populations. Further, as machine learning and artificial intelligence tools are increasingly applied to EHR data with the goal of driving medical innovation, these biases risk being encoded into analytic tools, potentially producing models that perform poorly, or even incorrectly, for populations that are underrepresented or mischaracterized in the underlying data.

Taken together, these findings highlight the need to more explicitly account for inequity when assessing EHR DQ. The biases we identified in completeness and concordance measures suggest that the definition of bias as an independent dimension of DQ in a recent literature review may be insufficient for capturing biases across all dimensions of DQ.^10^ Instead, we suggest that bias should be considered an independent “axis” of EHR DQ complementing the existing dimensions and methods. Because EHR systems are codependent with the healthcare system in which they operate, many facets of EHR DQ are unsurprisingly plagued by biases reflecting causal drivers of EHR data collection. Although we focused on bias related to systemic causes of inequity in healthcare access, biases in EHR DQ reflecting healthcare needs or health system practices also exist.^40–42^ Recognizing bias as a core axis of DQ can guide targeted improvement efforts by distinguishing issues rooted in healthcare system-level documentation practices or clinical developments from those reflecting population-level barriers to care. This differentiation enables more targeted interventions, that is, improving data collection processes where needed while addressing structural barriers to care when disparities reflect patterns in access rather than documentation. It can also help differentiate reasonable data gaps, such as absent laboratory results among healthy patients and relevant clinical changes, from problematic patterns indicating exclusion or inconsistent documentation. From a research perspective, quantifying bias enables investigators to evaluate how DQ issues influence secondary analyses and to design methods that mitigate these effects. Establishing standardized and consistent bias assessments across clinical domains will be essential for advancing EHR DQ, enhancing care provision, and improving the validity of data-driven research.

Conceptualizing bias as an axis of EHR DQ further enables the development of generalizable interventions and policy-relevant strategies to strengthen real-world data sources across reuse settings. Bias-aware DQ assessment can be used to identify populations for whom DQ is systematically lower, motivating provider- or system-level efforts to improve data capture and documentation practices. In the context of pregnancy dating, for example, such efforts might include standardizing EDD documentation workflows or implementing reconciliation checks when multiple EDDs are recorded. Second, identifying populations with persistently low-quality data can inform targeted outreach to better understand barriers to accessing care that contribute to both data gaps and inequitable health outcomes. In maternal health settings, this may involve examining how late entry into prenatal care or care fragmentation across institutions affect the availability and consistency of EDD data. Finally, incorporating bias-aware DQ metrics into research practice supports transparent reporting of how DQ-driven inclusion and exclusion criteria shape real-world evidence cohorts or predictive model development.^43–45^ For studies relying on EDDs to define pregnancy timelines, this transparency clarifies how missing or discordant EDDs influence cohort composition and downstream inferences. Together, these approaches position bias-aware DQ assessment as a broadly applicable framework that can be adapted to specific clinical contexts while improving equity, validity, and accountability in EHR-based research, ultimately bridging existing gaps between independent assessments of bias in EHR data more generally and post hoc bias assessments in artificial intelligence.^46^^,^^47^

### Limitations

There are many more variables for which DQ could have been assessed in this study. We were only able to incorporate a limited set of analyses, specifically those related to EDD. Other variables of interest could include the presence of an ultrasound during weeks 18-22 of gestation. A more extensive and automated pipeline for evaluating DQ in the context of prenatal care could strengthen this work and enable broader insights into data integrity across different domains.

We were also unable to determine the objective causes of the observed DQ issues. While we can speculate based on clinical expertise and existing literature, we cannot conclusively identify the origins of these patterns. We did not test for interactions between race and location leaving us further unable to distinguish between individual and regional features as drivers of observed biases. The causation challenge is exacerbated by the fact that we used data from a single health system in a region with multiple large health systems. The dynamic and fragmented nature of healthcare access, particularly during the prenatal care and delivery processes, may result in missing information in one system that is well documented in another. Further, residential segregation and associated socioeconomic divides in St. Louis may also drive the spatial DQ challenges through healthcare access and utilization patterns. Separating the effects of healthcare utilization and access patterns from health system related documentation errors on spatial bias would require data sharing efforts across health systems to distinguish between data sharing challenges, lack of healthcare access, and documentation practices.

## Conclusion

The goal of this project was to assess DQ of EHR data collected in the prenatal care setting and identify potential biases in quality levels. By using EHR data from a large health system in Saint Louis, MO and external data sources, we identified quality issues and bias in quality issues in the completeness and concordance of EDDs. We ultimately identified a need for exploring biases in EHR data that may differentially impact regions and communities. In prenatal care, we recommend standardizing EDD reconciliation workflows including documentation of the reasons for EDD changes (ie, new clinical findings or data entry errors). Further, we recommend building bias-aware DQ metrics into EHR-based prenatal cohort construction to ensure biases are reported and addressed in downstream efforts in. More broadly, measuring and reporting bias in EHR DQ should be a central objective across EHR data reuse projects to transparently characterize sources of biases and discuss their potential downstream impacts, and across health systems to inform future efforts focused on improving EHR DQ and reducing inequities in secondary data use. Addressing these areas in future work will improve the quality, comprehensiveness, and utility of EHR data, enhancing patient care and advancing medical research.

## Data Availability

The data underlying this article cannot be shared publicly to protect patient privacy. The data will be shared on reasonable request to the corresponding author.
